# Synchrotron radiation-based experimental determination of the optimal energy for cell radiotoxicity enhancement following photoelectric effect on stable iodinated compounds

**DOI:** 10.1038/sj.bjc.6601951

**Published:** 2004-07-13

**Authors:** S Corde, A Joubert, J F Adam, A M Charvet, J F Le Bas, F Estève, H Elleaume, J Balosso

**Affiliations:** 1 INSERM U647 ‘Rayonnement Synchrotron et Recherche Médicale’, Université Joseph Fourier & ID17 Biomedical Beamline of European Synchrotron Radiation Facility, CHU A Michallon, BP 217, 38043 Grenoble Cedex 09, France; 2 Unité IRM, service de Neuroradiologie, CHU A Michallon, BP 217, 38043 Grenoble Cedex 09, France; 3 IFR no. 1 ‘RMN biomédicale, de la cellule à l'homme’, CHU A Michallon, BP 217, 38043 Grenoble Cedex 09, France; 4 Département de Cancérologie et d'Hématologie, Service de Radiothérapie, CHU A Michallon, BP 217, 38043 Grenoble Cedex 09, France

**Keywords:** monochromatic synchrotron radiation, radiotherapy, iodine, radiation dose enhancement

## Abstract

This study was designed to experimentally evaluate the optimal X-ray energy for increasing the radiation energy absorbed in tumours loaded with iodinated compounds, using the photoelectric effect. SQ20B human cells were irradiated with synchrotron monochromatic beam tuned at 32.8, 33.5, 50 and 70 keV. Two cell treatments were compared to the control: cells suspended in 10 mg ml^−1^ of iodine radiological contrast agent or cells pre-exposed with 10 *μ*M of iodo-desoxyuridine (IUdR) for 48 h. Our radiobiological end point was clonogenic cell survival. Cells irradiated with both iodine compounds exhibited a radiation sensitisation enhancement. Moreover, it was energy dependent, with a maximum at 50 keV. At this energy, the sensitisation calculated at 10% survival was equal to 2.03 for cells suspended in iodinated contrast agent and 2.60 for IUdR. Cells pretreated with IUdR had higher sensitisation factors over the energy range than for those suspended in iodine contrast agent. Also, their survival curves presented no shoulder, suggesting complex lethal damages from Auger electrons. Our results confirm the existence of the 50 keV energy optimum for a binary therapeutic irradiation based on the presence of stable iodine in tumours and an external irradiation. Monochromatic synchrotron radiotherapy concept is hence proposed for increasing the differential effect between healthy and cancerous tissue irradiation.

Megavoltage radiation beams are nowadays widely used for anticancer radiotherapy. High-energy linear accelerators allow highly penetrating radiation to treat targeted volumes with a better sparing of superficial healthy tissues. Moreover, in this range of energies (1–25 MeV), photons beams mainly interact with living matter by Compton scattering processes, almost independent of the composition of the traversed material. Thus, a highly homogeneous dose distribution is achievable in the human body and predicted accurately by Monte-Carlo based three-dimensional treatment planning systems.

Lower energy beams are preferred for medical imaging. Kilovoltage X-ray beams are used for the production of radiological images and the predominant physical process in high-density materials is the photoelectric effect, which is strongly Z-dependent. Hence, attenuation in bone is far higher than for soft tissues, and the radiological contrast rely on photoelectric processes. The spectra of these X-ray tube beams vary between 10 and 250 keV.

Healthy tissue tolerance remains the major limiting factor of anticancer radiotherapy and techniques are investigated to enhance the local control of tumours by increasing the absorbed X-ray dose, while preserving more efficiently the healthy surrounding tissues.

Dual modalities like Boron Neutron Capture Therapy (BNCT) propose enhancing the neutron capture reaction cross-sections in a stable boron-loaded tumour (Barth *et al*, 1990). Toxicity of the irradiation is increased in the tumour by the production of high linear energy transfer alpha and lithium ion particles. The neutron dose enhancement is highly correlated to the boron concentration present inside the tumour at the time of the irradiation. The most limiting factor remains the boron concentrations achievable (Soloway *et al*, 1994; Coderre *et al*, 1998) and their *in vivo* measurement (Verbakel and Stecher-Rasmussen, 2001). Nevertheless, clinical trials are underway for patients with high-grade brain gliomas, which remains a hardly curable tumour type.

For photon beams, a bimodal approach known as ‘Photon Activation Therapy’ has been proposed in the early 1970s (Tisljar-Lentulis *et al*, 1973; Tisljar-Lentulis and Feinendegen, 1977). High-Z heteroatoms in DNA could be used for increasing kilovoltage X-ray killing efficiency, by generating photoelectric events. Ionised heavy atoms then reorganise and emit low-energy Auger electrons cascades, able to damage DNA because of their nuclear localisation. In a less specific way, Norman *et al* developed another approach: the use of a conventional kilovoltage scanner to treat iodine-loaded tumours (Iwamoto *et al*, 1990; Rose *et al*, 1999). By increasing the tumour density and focussing a kilovoltage beam on it, interaction cross-sections are improved in the tumour only, due to the photoelectric effect, and the dose distribution is concentrated inside the tumour (Solberg *et al*, 1992a, 1995; Mesa *et al*, 1999). Moreover, tumour imaging is achievable during treatment with this scanner. The lack of such a modality is actually a weakness of current megavoltage radiotherapy treatments. The physical properties of high-energy beams allow good dose distribution characteristics but do not allow good imaging yet.

What would be the optimal X-ray energy, if any, for irradiating tumours loaded with high-Z elements? Considering a heavy element introduced to living matter as isolated atoms, irradiation with an energy just above the K-edge appears conceptually as the best option to enhance energy deposition by the contribution of Auger electrons events. This concept has been tested with iodine and other heavy atoms, irradiated with either monochromatic radioactive sources (Nath *et al*, 1990), or synchrotron X-rays (Laster *et al*, 1993; Hieda *et al*, 1996). Laster *et al* obtained convincing results with iodine incorporated into DNA as 5-iodo-2′-deoxyuridine (IUdR). Others, as Solberg *et al* (1992a) and Karnas *et al* (1999) suggested, by calculation, that the optimal energy should be far above the iodine K-edge and around 50 keV because it corresponds to the energy level where the difference between the mass energy absorption coefficients of water and iodine is the largest. Therefore, two different concepts were to be considered: the first one based on the occurrence of particular events; the second one taking into account the difference in absorption capacity of the living matter with and without the presence of a certain concentration of iodine, whatever the structure imbedding it.

As pointed out by Karnas *et al* (1999) and Solberg *et al* (1992b), monochromatic X-rays produced by a synchrotron should be the best tool for verifying these hypotheses. Powerful third generation synchrotrons are now available to deliver broad X-ray beams (10–100 keV), brilliant enough for using monochromators and able to precisely select the desired X-rays energy. At the European Synchrotron Radiation Facility (ESRF, Grenoble, France), the ID17 beamline is dedicated to medical applications of synchrotron radiation (Thomlinson *et al*, 2000) and is designed for human *in vivo* experiments (Elleaume *et al*, 1999, 2000a).

In this paper we report the experimental demonstration of the existence of an optimal value of the enhancement ratio produced by the presence of iodine compounds. We studied its variation along the energy spectrum from 30 to 70 keV in two different experimental settings: extracellular iodine from contrast agent and iodine incorporated into DNA as IUdR.

## METHODS AND MATERIALS

### Chemicals

5-iodo-2′-deoxyuridine was provided by Lausanne University Hospital Pharmacy (Switzerland) as lyophilised powder. Iodinated radiographic contrast agent used for extracellular iodine was Iomeron® (Bracco, Milano, Italy), which is a nonionic, monomeric iodine compound containing 350 mg of iodine per ml of solution.

### Cell line

The SQ20B cell line was derived from a human head and neck squamous carcinoma (Weichselbaum *et al*, 1986) and was obtained from William K Dahlberg (Dr JB Little Laboratory, Harvard School of Public Health). This cell line is commonly used for radiobiological research, and has been handled as previously described (Corde *et al*, 2002). The SQ20B doubling time is 21 h.

### Cell culture technique, colony-forming assay

Cells were seeded and grown as monolayer in plastic tissue culture disposable flasks (Falcon) with 0.4 ml cm^−2^ Dulbecco's modified Eagle's minimum medium (Gibco-BRL), added with 10% foetal calf serum (Gibco-BRL), penicillin and streptomycin (Gibco-BRL). Cells were grown at 37°C in a humidified atmosphere of 5% CO_2_ in air.

When IUdR was used, 48 h before irradiation, cell cultures were incubated with 10 *μ*M IUdR (1.3 mg iodine l^−1^), diluted in fresh medium. Before irradiation, cells were trypsinised and experiments were carried out with the cell suspension in 2 ml sterile cryotubes (Merck Eurolab) with or without Iomeron® (10 g iodine l^−1^) added to the culture medium, according to the experimental protocol.

Three independent experiments have been carried out and averaged. After each irradiation, triplicate low-density subcultures of the cells were established in ∅100 mm Petri dishes for colony-forming assay. Colonies were fixed and stained with violet crystal oxalate (Merck Eurolab) after 15 days of cell growing.

### Irradiation procedure

Irradiation was carried out at room temperature in aerobic conditions at the ESRF medical beamline (ID17) (Elleaume *et al*, 1999). Cells were irradiated as suspension in horizontal continuously rotating cryotubes (2 ml, 10 mm in diameter), vertically translated up-and-down through a 500-*μ*m-thick X-ray beam providing a dose rate calculated in water of about 0.5 Gy s^−1^. Dose calibrations were performed using a cylindrical ion chamber (PTW 31002) coupled with a Unidos® electrometer. They were cross-checked with a high-purity Germanium detector (Eurisys Mesures®, Lingolsheim, France). Real-time control of delivered doses was provided by a 10 cm long nitrogen filled ion chamber, continuously present in the beam.

Energies were tuned with an accuracy of ±100 eV. The dose rate was equal for all the tested energies, which were 32.8, 33.5, 50 and 70 keV. We point out that the K-edge of iodine is 33.169 keV. This setting was obtained with an Si(111) fixed-exit monochromator, designed for computed tomography with synchrotron radiation as previously described (Suortti *et al*, 2000; Corde *et al*, 2002). The ESRF storage ring was operating in its ‘uniform mode’, providing a storage ring current decreasing from 200 to 170 mA with a lifetime of 60 h.

### Data analysis

Colonies with more than 50 cells were counted using digital images of Petri dishes containing fixed and stained clones, associated with a computer assisted image analysis system (Samba Technologies, Meylan, France) as previously described (Biston *et al*, 2003), which allows to avoid subjective bias in the procedure.

Experimental data were fitted with the linear quadratic model (LQ):


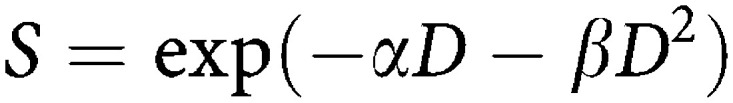


where S is the survival probability, D the radiation dose (Gy), *α* and *β* are the fit parameters (Gy^−1^ and Gy^−2^ respectively).

### Enhancement ratio

We used two different ratios to measure the dose modifications produced by the iodine.

The sensitisation enhancement ratio at ‘*S*%’ of survival, SER_s%_ was calculated from the experimental cell survival curves and defined as the ratio of doses (sensitised to control) that yielded a given cell survival level of *S*%:


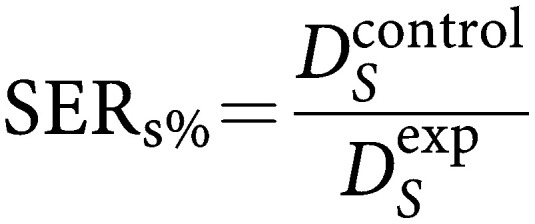


The theoretical expected dose enhancement ratio, DER, was calculated from the variation of the mass energy-absorption coefficient of the target due to the presence of iodine:


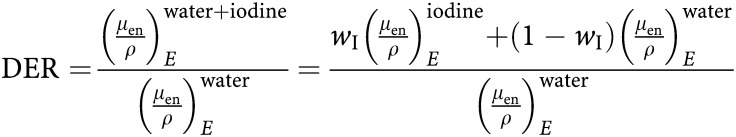


where


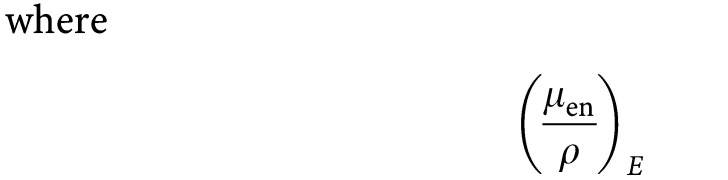


is the mass energy absorption coefficient for the considered compound irradiated with monochromatic X-rays beam (energy: *E*) and *w*_I_ is the fraction by weight of iodine in the mixture.

## RESULTS

### Energy dependence of the DER for different iodine concentrations in water

**Figure 1:**
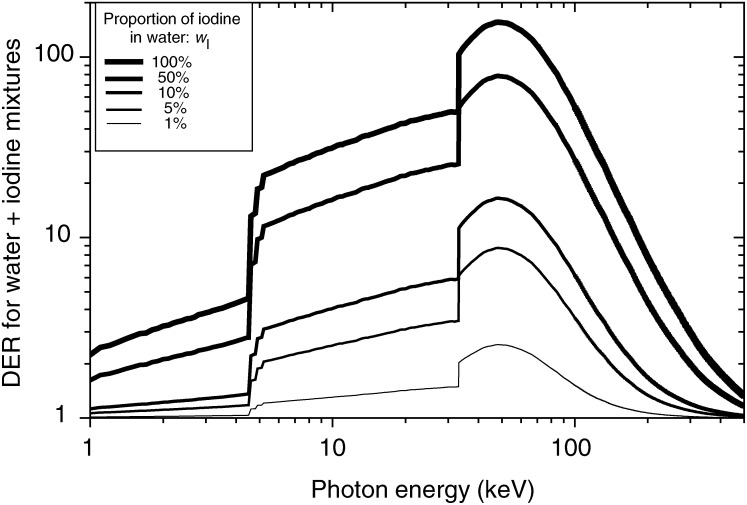
Energy dependence of the theoretical dose-enhancement ratio for several iodine aqueous mixtures (from bottom to top, the mass proportion of iodine in water, *w*_I_ is ranging from 0.01 to 1).

DER variation with photon energy has been represented in Figure 1

from Hubble physical data references (Hubbell and Seltzer, 1997). This variation is a bell-shaped curve having its maximum around 50 keV. The maximum DER is strikingly increased when the iodine concentration in water is rising up to 100%, reaching about 160. The range of energies yielding such a sharp variation is rather narrow from the K-edge of iodine (33 keV) up to about 80 keV.

### Experimental energy dependence of the SER for cells irradiated with 10 mg ml^−1^ of extracellular iodine in medium

**Figure 2:**
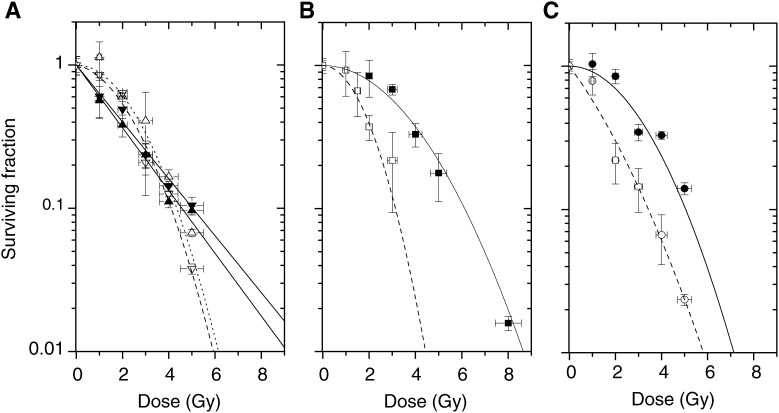
Survival curves of SQ20B cells irradiated with (open symbols) or without (closed symbols) 10 mg ml^−1^ iodine incorporated as contrast agent in medium, for the energies: (**A**) around iodine K-edge: 32.8 keV (triangles) and 33.5 keV (reversed triangles); (**B**) 50 keV (squares); (**C**) 70 keV (circles). Each survival curve fit is derived from the dose and surviving fraction data (triplicate experiments).

Survival curves of SQ20B cells are displayed in Figures 2A–C

, showing the modifications of the dose–effect relationship for synchrotron radiation of different energies with or without 10 mg ml^−1^ of iodine introduced as Iomeron®. These experimental results have been obtained with a constant dose rate whatever the energy level. It appears that the radiosensitisation is energy dependent.

**Figure 3:**
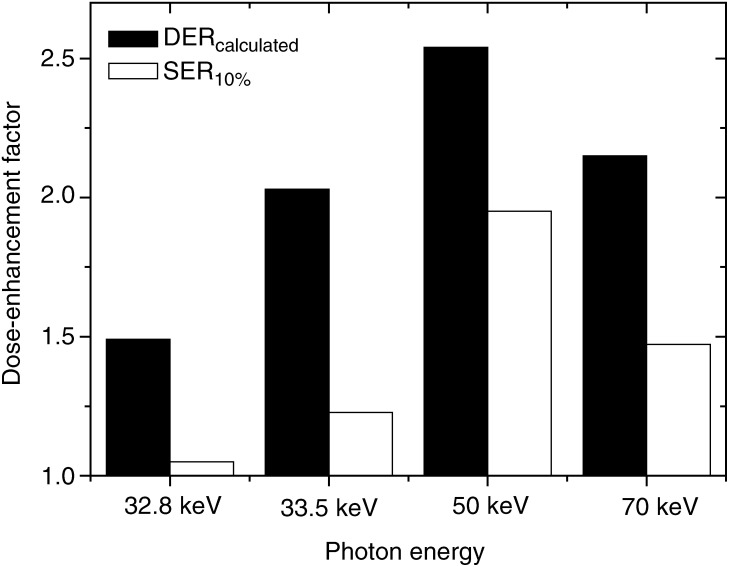
Comparison of the energy dependence of the calculated dose-enhancement factor (DER) with the measured one at 10% survival level (SER_10%_), for 10 mg ml^−1^ of extracellular iodine.

The comparison of experimental SER_10%_ with the calculated DER, according to the energy, is shown in Figure 3

. The variation in the behaviour of both parameters with energy is similar with the same apparent maximum at 50 keV but experimental results are constantly lower than predicted. The experimental SER_10%_ increases slightly when the energy goes through the iodine K-edge (from 1.05 to 1.23), but reaches its maximum for 50 keV (1.95) and decreases again for 70 keV as the theory predicts.

### Experimental energy dependence of the SER for cells irradiated with 10 *μ*M of iodine incorporated in DNA as IUdR

**Figure 4:**
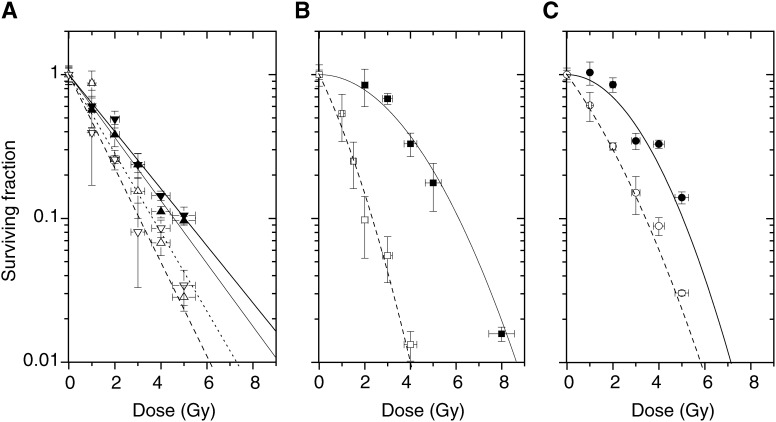
Survival curves of SQ20B cells irradiated with (open symbols) or without (closed symbols) a 48-h pre-exposure to 10 *μ*M IUdR, for the energies: (**A**) around iodine K-edge: 32.8 keV (triangle) and 33.5 keV (reversed triangle); (**B**) 50 keV (square); (c) 70 keV (circle). Each survival curve fit is derived from the dose and surviving fraction data (triplicate experiments).

The same experiment was performed with iodine incorporated into the cell nucleus as iodinated nucleotide by exposure to 10 *μ*M IUdR during 48 h followed by irradiation at different energy levels. Once again, we observed a cell sensitivity energy dependence, for the same dose rate of monochromatic irradiation, as shown in Figures 4A–C

. Similarly, 50 keV is the energy yielding the maximum cytotoxic effect. The experimental SER_10%_ obtained is 2.6 for 50 keV, and the difference below *vs* above the K-edge of the iodine is fairly reduced, the values are, respectively, 1.25 and 1.64.

### Comparison of the effects of both iodine compounds

Figure 5

**Figure 5:**
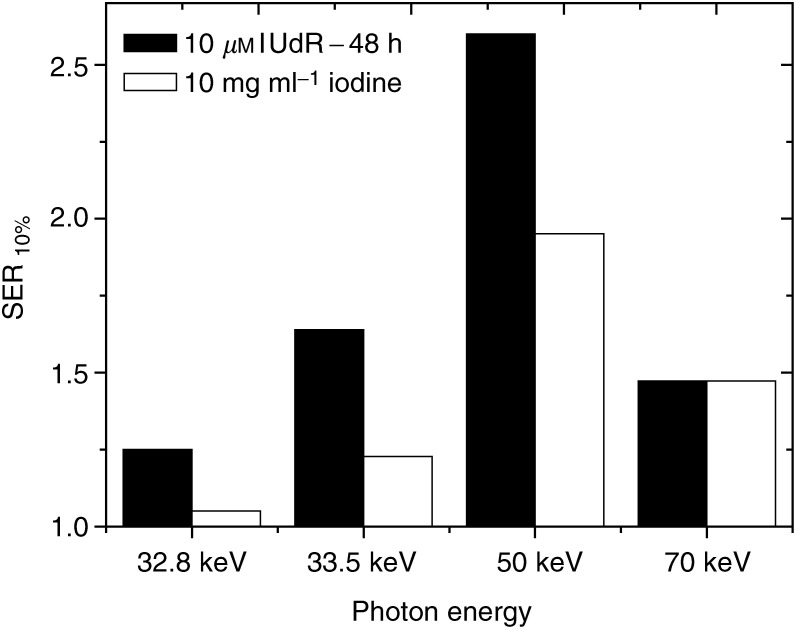
Comparison of the energy dependence of the sensitization enhancement ratio (SER_10%_) for cells pretreated with 10 *μ*M IUdR for 48 h or irradiated with 10 mg ml^−1^ of iodine incorporated in their medium as contrast agent.

compares the SER_10%_ obtained with SQ20B cells irradiated at different energies with either 10 mg ml^−1^ extracellular iodine or intracellular iodine incorporated into the nucleus as IUdR. For the same survival level, namely 10%, the intracellular situation of iodine proved to be more efficient than the extracellular case except for the highest energy of 70 keV.

**Figure 6:**
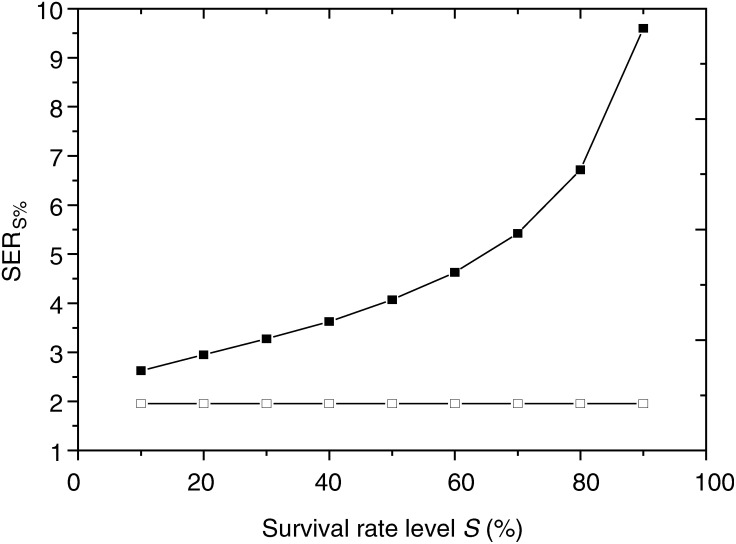
Comparison of the survival level dependence for the calculation of the experimental factor SER_S%_ for cells irradiated at 50 keV either with a 48 h pre-exposure to 10 *μ*M IUdR (closed symbols) or with 10 mg ml^−1^ iodine incorporated in the medium via a contrast agent (open symbols).

Interestingly, the comparison of survival curves from Figure 2 and 4 shows a more deeply altered shape for intracellular iodine than for the extracellular iodine. The initial shoulder is strongly reduced giving a high-TEL-radiation-like shape for intracellular iodine. As an illustration, Figure 6

shows the variation of the SER_s%_, according to the survival level, measured at 50 keV. A striking difference is observed between both iodine situations. For intracellular iodine, the sensitisation enhancement ratio increases from 2.6 for a survival rate of 10% up to almost 10 for 90% survival. For the extracellular iodine, the sensitisation enhancement ratio is almost constant around 2.

## DISCUSSION

The sensitivity of SQ20B cells to kilovoltage X-ray beams is enhanced in the presence of the iodine compounds, whatever its subcellular localisation. This enhancement depends upon the choice of the X-ray energy beam, as predicted by the theoretical DER curves (Figure 1).

### Iodine contrast agent as radiation sensitiser

When extracellular iodinated contrast agent was used, experimental values of the enhancement ratio, estimated at 10% of the surviving fraction from the linear-quadratic interpolation of the survival curves, was systematically inferior to the DER by a factor varying between 23% (50 keV) to 40% (33.5 keV).

Such differences between the calculated and the experimental enhancement ratio could be explained by different factors:

Since not all energy deposited results in cell killing, the theoretical dose enhancement ratio will always overestimate *in vitro* or *in vivo* measurements.
The experimental working conditions are arbitrary (choice of the cell line, geometry of the setup, cell density during irradiation).
Calculation of the theoretical factor does not take into account the experimental geometry: a physical autoabsorption of the photoelectrons by the medium itself and as a consequence less radiation dose seen by the cells in suspension in their medium could be taken into consideration by finer simulations.
Finally, the survival rate level chosen for the comparison is of prime importance as suggested in Figure 6.

Nevertheless, the shape of both the enhancement ratio variations are consistent, with an experimental maximum at 50 keV and a minimum just below the iodine K-absorption edge (Figure 3). Moreover, this enhanced radiosensitivity is concentration dependent, as published elsewhere (Estève *et al*, 2002).

We interpret the lethal effect enhancement observed as closely linked to photoelectric interaction on the high-Z atoms present in the vicinity of the SQ20B cells (Callisen *et al*, 1979). As studied by Matsudaira *et al* (1980), the use of more energetic radiation, such as *γ* for instance, would not imply similar results. With these energies comparable with the ones used in radiotherapy, Compton scattering is the major physical interaction and does not vary sufficiently with the matter composition for giving probing enhancement factors (Robar *et al*, 2002). Matsudaira *et al* (1980) demonstrated with a 200 kV_p_ polychromatic (p) X-rays tube that 5% of iodine in the cell growing medium modified cellular response to the irradiation, but no effect was shown with irradiation from ^60^Co.

We extrapolated their survival curve data and estimated that the SER_10%_ factor was equal to 2.24 for 50 mg ml^−1^ of iodine and 200 kV_p_ irradiation. Dawson *et al* (1987) have found similar results with an SER_10%_ estimated from their data to be around 1.8 for 20 mg ml^−1^ of iodine and 250 kV_p_ irradiation. Interpolation of their data to 10 mg ml^−1^ of iodine would have led to a sensitisation enhancement ratio of 1.3. This factor has been increased to 2.25 by using a lower energy X-ray beam of 140 kV_p_ (Iwamoto *et al*, 1987; Solberg *et al*, 1992a). Nevertheless, this last value has not been calculated from survival curves data, but from micronuclei formation in cells postirradiation.

These experimental demonstrations of the radiotoxicity enhancement of low- and medium-energy X-rays due to the presence of iodinated contrast agents had some consequences in radiological diagnosis, mainly because of the fear of mutagenic effects postexamination (Adams *et al*, 1977; Norman *et al*, 1978, 2001). Nevertheless, the X-ray doses implied in such examinations are not comparable with the therapeutic ones.

Based on these results, a brand new radiotherapy technique called CTRx for computed tomography radiotherapy was put forward by Norman and collaborators. They propose using a classical scanner (voltage 140 kV_p_) slightly modified for allowing field collimation, adjustable to tumour sizes. The aim of the technique is to obtain sharp isodoses around the tumour, using both the photoelectric effect on the high-Z element present in the tumour and the circular irradiation ballistic (Mesa *et al*, 1999). Phase I clinical trial was published for treatment of patients with metastatic brain tumours, loaded with iodinated contrast agents, and demonstrated the feasibility of this technique (Rose *et al*, 1999). The dose-enhancement effect is hence theoretically optimised with 140 kV_p_ X-rays beams when compared with 10 MV, and, interestingly enough even in cases of stereotactic irradiation.

However, polychromatic conventional X-ray tubes do not lead to an optimum energy deposition inside the iodine-loaded tumour (X-ray spectrum hardening with depth). Synchrotron X-rays beam should allow the choice of the optimal X-ray energy for energy absorption enhancement inside the iodinated tumour compared with healthy nonloaded tissues (Solberg *et al*, 1992b).

Our results show that this optimal energy does exist and according to our calculations, its value is around 50 keV. For this particular energy, the SER_10%_ measured on our cell line is 2.03 for 10 mg ml^−1^ of iodine incorporated as contrast agent in the medium. This sensitisation value appears to have the same order of magnitude for monochromatic beam in comparison with the results quoted above with polychromatic beams. Nevertheless, such comparisons have to be made carefully and are always difficult when different cell lines are considered. It should be stressed that SQ20B cell line is particularly radioresistant and nonapoptotic (Brachman *et al*, 1993).

Another advantage of the synchrotron radiation beam, for this bimodal approach, is the imaging scanner tool, which was developed on the ESRF ID17 medical beamline (Elleaume *et al*, 2000b, 2002). It allows the *in vivo* measurement of absolute tumoral iodine concentrations (Le Duc *et al*, 2000), which is an essential parameter for planning the doses to be delivered. This advantage is not available with other bimodal approaches: neither with conventional scanners due to beam hardening nor for BNCT as previously mentioned. This could be a valid argument for considering this technique for clinical trial evaluation.

### Iodine incorporated into DNA as IUdR: subcellular and energetic optimisations

5-iodo-2′-deoxyuridine was used in this study to optimise the subcellular localisation of iodine compounds. 5-iodo-2′-deoxyuridine does not remain extracellular as contrast agents, but directly substitutes DNA thymidine base, and is hence incorporated inside the DNA morphology during its replication in cycling cells (Pomplun and Terrissol 1994). This compound is known to sensitise mammalian cells to damage induced by ionizing radiation, both *in vitro* and *in vivo*. The underlying sensitisation mechanism is not yet fully understood but could rely on alteration of the DNA structure (Iliakis and Kurtzman, 1989) or a decrease in cell reparability (Wang and Iliakis, 1992; Wang *et al*, 1994). As this compound requires a cellular proliferation activity, it has the property to target tumours having high proliferation rates.

The photonic activation of stable iodine atoms, incorporated in DNA with IUdR, was first proposed by Fairchild *et al* (1982). They calculated a sensitisation factor comprised between 1.5 and 3 depending on the percentage of thymidine replacement. This technique avoids the drawbacks of IUdR labelled with Auger emitters radioisotopes, which are also toxic for healthy fast-growing tissues such as the bone marrow.

Whereas clinical use of IUdR in association with megavoltage X-rays beams brought to reserved results (Epstein *et al*, 1992), photon activation therapy proposed by Fairchild and Bond (1984) was based not only on IUdRs use but also on the idea of an energetic optimisation of the X-ray beam too. Photoelectric absorption discontinuities of the isolated heavy atoms lead to the conclusion that the energy just above the K-absorption edge of iodine was the optimal one. Laster *et al* (1993) demonstrated, for this particular energy, that the dose necessary to decrease the surviving V79 cells fraction to 10% was three times less for cells pre-exposed to 6 *μ*M IUdR for 14 h (16% substitution) than for the untreated cells. This value decreased to 1.4-fold less when synchrotron X-rays energy was just below the iodine K-edge. These are the most satisfying results yet, among a series of similar works, which had common objectives but used different experimental methods. A sensitisation ratio equal to 1.8 has been observed by Shinohara *et al* (1996) with 20% thymidine bases substituted by iodine from IUdR and a γ-irradiation from ^60^Co. Another study with ^137^Cs irradiation and similar iodine content gave comparable results (Fairchild *et al*, 1985). This factor was found to be equal to 1.5 by Nath *et al* (1987) with 250 kVp X-rays.

The choice of the optimal X-ray energy for irradiating IUdR pretreated cells is investigated in the present work, similarly to Karnas *et al* (1999), (2001a). Theoretical calculation of the DER relative to the presence of 10 *μ*M of IUdR during the irradiation in water would be negligible, very close to 1, except if this factor is calculated at the microscopic level, as suggested by Karnas *et al* In that case, a DER comprised between 2 and 3 for 50 keV irradiation is expected for a 10–30% thymidine base replacement by iodine. Other authors have reported comparable measured DNA iodine contents for exposure of exponentially growing cells. Hence, for a 10 *μ*M IUdR during a double cell cycling time (48 h), a thymidine substitution ranging between 10 and 20% is expected (Laster *et al*, 1993).

Consequently, our experimental results are in good agreement with the calculations made by Karnas *et al* (1999). Due to the lack of available synchrotron sources, adjustable monochromatic beams were not used to validate their calculation, but CHO cells exposed to 10 *μ*M IUdR for 3 days and irradiated with medium energy X-ray tube (100 kV_p_) exhibited SER_1%_ of 1.8 *vs* 1.4 for low-energy spectrum (30 kV_p_). By filtrating the 100 kV_p_ beam with tungsten, SER_1%_ reached a value of 2.7, corresponding to a thymidine substitution by iodine of 18%.

With a monochromatic beam, SER_10%_ for SQ20B cells pre-exposed for 48 h to 10 *μ*M IUdR was found to be equal to 2.62 at 50 keV. This energy corresponds to the experimental maximum of radiosensitisation. Interestingly, around the K-edge of iodine, SER_10%_ slightly increases by crossing the edge (from 1.25 to 1.64) but our enhancement ratios remain low in comparison with some other published data (Laster *et al*, 1993). Moreover, energies around the K-edge are experimentally found not to be optimal for increasing the differential effect between treated and untreated cells.

Owing to the energy dependence of the sensitisation found for cells pretreated with IUdR, it is more likely due to the photoelectric effect than to an intrinsic sensitisation action of IUdR, for which an energy dependence could hardly be expected.

### Comparison of the effects of both iodine compounds

Pre-exposure of cells to 10 *μ*M IUdR for 48 h leads to greater sensitisation enhancement ratios than the presence of 10 mg ml^−1^ of iodine as contrast agent in the medium (Figure 5). 5-iodo-2′-deoxyuridine is 30% more efficient at 33.5 and 50 keV, 20% more at 32.8 keV and sensitisation ratios appear to be the same at 70 keV. Above the K-edge of iodine, photoelectrons from the K shell are extracted with a kinetic energy of 300, 18.8 and 36.8 keV for 33.5, 50 and 70 keV excitation energies, respectively. Reported to the nucleus and cellular dimensions, the range of photoelectrons emitted by iodine atoms placed inside the DNA is then less critical for the highest energies studied. The biological efficiency of photon activated IUdR with regard to the extracellular iodine could be explained by damages produced directly inside the DNA by Auger electrons. These low-energy electron cascades are known to be biologically efficient and extremely toxic, but only if generated in the nucleus or preferably in the DNA (Commerford *et al*, 1980; Faraggi *et al*, 1994; van Dieren *et al*, 1996).

The effect of Auger electron cascades for IUdR can be deduced by analyzing the shape of survival curves (Figures 4 and 6). The absence of a shoulder indicates a defect in repair of DNA damages, which are supposed to be more complex. On the contrary, survival curves for iodine incorporated as contrast agent do not show any loss of shoulder and seem to indicate a simple creation of an extra number of both single strand breaks (SSB) and double-strand breaks (DSB). For IUdR data, this leads to an extreme dependence of the SER factor with the choice of the survival level *S*% used for the calculation. The SER_90%_ reaches a value of 9.5 around 90% of cell survival rate, which could be worth considering for a fractionated irradiation with low dose per fraction. On the contrary, the ratios remain similar for iodinated contrast agent, whatever the survival level chosen. The survival curves exhibit then low-LET shapes, indicating that in both approaches, completely different biophysical and molecular damages are generated inside the DNA.

Some reserve has been expressed in the literature concerning the potential clinical use of externally synchrotron-activated stable IUdR (Miller *et al*, 1987a, 1987b; Humm, 1988a; Humm and Charlton 1988b). Our encouraging results could certainly boost the debate. The two new elements in the discussion could be the relative effects of both the iodinated compounds and the choice of the optimal energy to increase the enhancement ratios.

Other clinical applications could be found by associating a monochromatic photon source with a heavy intratumoral element. The ideal radioisotope would be one able to optimise interactions with the targeted heavy atom. Of course, this limits the number of (emitter–receptor) couples to be considered, but opens the way to new source concepts (Karnas *et al*, 2001b). Samarium sources, which emit monochromatic 40 keV *γ* photons, could be used, for instance, in association with stable iodine (Fairchild *et al*, 1987; Laster *et al*, 1992). Energy deposited by ^125^I seeds during prostate brachytherapy treatment could be enhanced by silver compounds (Young *et al*, 1999) or other contrast agents like iodine, gadolinium or lutetium as well (Norman *et al*, 2002).

However, the synchrotron tool appears to be the most useful one for optimizing such a concept of bimodal radiotherapy and monochromatic synchrotron radiotherapy, which exhibits promising *in vivo* results (Adam *et al*, 2003). This concept is hence proposed as a promising technique for optimally increasing the differential effect between healthy and cancerous tissue irradiation.
